# Microwave Detection of Carbon Monoxide Gas via a Spoof Localized Surface Plasmons-Enhanced Cavity Antenna

**DOI:** 10.3390/mi16070790

**Published:** 2025-07-02

**Authors:** Meng Wang, Wenjie Xu, Shitao Sun

**Affiliations:** 1 Wuxi Campus, Southeast University, Wuxi 214127, China; 213220503@seu.edu.cn (W.X.); 220241219@seu.edu.cn (S.S.); 2 State Key Laboratory of Millimeter Waves, School of Information Science and Engineering, Southeast University, Nanjing 210096, China

**Keywords:** cavity resonance, carbon monoxide discrimination, remote detection, sensing antenna, spoof localized surface plasmons

## Abstract

This paper presents a carbon monoxide (CO) detection mechanism achieved through further improvement of the sensing antenna based on hybrid spoof localized surface plasmons (SLSPs) and cavity resonance. Unlike conventional approaches relying on chemical reactions or photoelectric effects, the all-metal configuration detects dielectric variations through microwave-regime resonance frequency shifts, enabling CO/air differentiation with theoretically enhanced robustness and environmental adaptability. The designed system achieves measured figures of merit (FoMs) of 183.2 RIU^−1^, resolving gases with dielectric contrast below 0.1%. Experimental validation successfully discriminated CO (ε_r_ = 1.00262) from air (ε_r_ = 1.00054) under standard atmospheric pressure at 18 °C.

## 1. Introduction

Carbon monoxide (CO), a colorless, odorless, and highly toxic byproduct of incomplete carbonaceous combustion, requires precise discrimination from ambient air. Current CO sensors—including electrochemical, semiconductor, infrared, catalytic combustion, and solid-electrolyte types—employ distinct operational principles [1,2,3]. While demonstrating sensitivity and cost advantages, they suffer from instability and poor tolerance to harsh environments (e.g., high temperature/humidity). In this case, this paper proposes a unique sensing antenna-based CO detection scheme. The sensing antenna is an improved design based on our recently proposed spoof localized surface plasmons (SLSPs)-enabled cavity antenna [4], whose all-metal configuration and simple structure theoretically ensure better environmental adaptability.

In the microwave, terahertz, and infrared frequency bands, SLSPs are the equivalent of naturally occurring localized surface plasmons (LSPs). LSPs naturally occur in the optical frequency band and are electromagnetic (EM) resonance effects triggered by mutual coupling between incident EM waves and free electrons within metal nanoparticles, exhibiting significant field enhancement effects [5,6,7]. However, when operating frequencies are below the infrared band, metals exhibit characteristics of perfect electric conductors (PECs). Under such circumstances, it is essential to fabricate periodic structures on the metal surface with dimensions significantly smaller than the wavelength. The generated localized electromagnetic waves formed in this process are referred to as SLSPs [8,9,10]. An intriguing feature is that SLSPs possess distinctive advantages of exceptional field confinement and enhancement capabilities. Additionally, their resonant frequencies demonstrate high sensitivity to variations in the material’s dielectric constant, which makes them an optimal choice for sensor applications [11,12,13,14,15,16,17]. However, no gas-detection-capable SLSP sensors have been reported due to sensitivity limitations.

Sensing antennas have emerged as a research focus [4,18] due to their dual capabilities in wireless detection and backhaul. It should be noted that while wireless sensing via plane-wave illumination of resonant surfaces is also feasible, such configurations inherently exhibit drawbacks of low Q-factor and sensitivity—direct consequences of their open-system architecture [19,20,21,22,23]. The antenna reported in Ref. [4] utilizes a hybrid mode combining SLSPs’ resonance and cavity resonance. Its all-metal design enables reliable operation in extreme environments [4]. However, although Ref. [4] achieves a higher Q-factor and sensitivity than conventional designs, its multi-slot configuration (for far-field-based higher-order mode suppression) remains inadequate for detecting minute dielectric variations between gases. Additionally, the compact probe feeding shows vulnerability to length errors. Therefore, it is unable to achieve the CO gas discrimination function.

In this work, by reducing the number of radiating slots to one and replacing probe feeding with a WR-187 waveguide, we developed a high-precision CO gas sensing system based on the previously proposed SLSPs-enabled cavity sensing antenna [4]. Specifically, a rectangular cavity and an SLSP resonator were combined to produce resonance effects that resulted in simulated and measured Q-factors of 522.1 and 474.3, respectively. Moreover, experimental results demonstrate a 4 MHz frequency shift when detecting 80 mL pure CO gas (1 atm, 18 °C) within the cavity, yielding a sensing figure of merit (FoM) of 183.2 RIU^−1^. In this way, the proposed sensing antenna can achieve high-precision remote discrimination between CO and air without relying on micro–nano structures or electrochemical devices. Its all-metal structure theoretically makes it more suitable for detection in extreme environments such as high temperature and high humidity.

## 2. Methods

**Figure 1:**
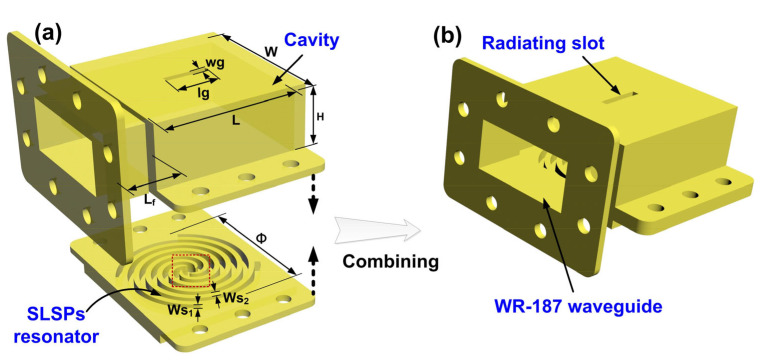
Schematic diagram of the proposed sensor antenna. (**a**) Structure diagram of the sensor antenna. (**b**) Assembled antenna.

Figure 1a,b show the proposed sensing antenna based on the hybrid resonant structure, which consist of the rectangular resonant cavity with a radiating slot and the SLSP resonator etched on a metal plane, both of which are tightly connected by six screws distributed on both sides. The geometrical parameters of the sensing antenna are as follows: *L* = 60 mm, *W* = 60 mm, *H* = 26.5 mm, *lg* = 18.8 mm, *wg* = 3 mm, *W*_s1_ = 2.4 mm, *W*_s2_ = 2.5 mm, Φ = 62 mm. A section of WR-187 standard waveguide of length *L_f_* = 20 mm is designed to guide the feeding EM waves into the cavity.

**Figure 2:**
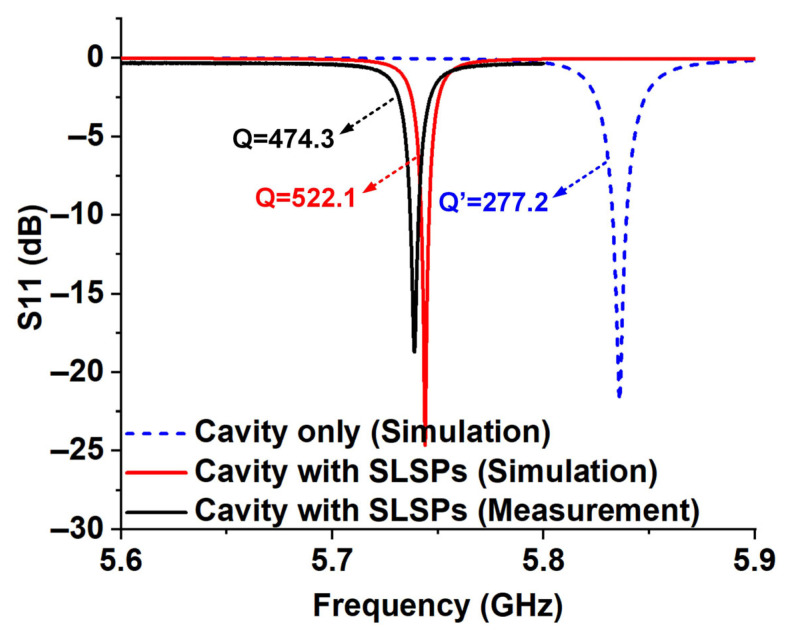
High Q-factor characteristics of the proposed sensor antenna.

According to our previous research, the significant advantage of this sensor antenna lies in its extremely high Q-factor achieved through the hybrid mode of spoof localized surface plasmons (SLSPs) resonance and cavity resonance [4]. The S-parameters of the proposed sensor antenna are shown in Figure 2, where the simulated and measured results are in good agreement except for slight frequency deviations caused by processing errors. The simulated and measured resonant frequencies of the antenna are 5.7435 GHz and 5.7389 GHz respectively, with calculated Q-factors of 522.1 and 474.3—values that are extremely high for an open antenna system. As a comparison, the simulated Q-factor of the sensing antenna without SLSP structure is only 277.2. Moreover, by reducing the number of radiating slots, the antenna’s Q-factor in this work is further improved even compared to the design proposed in Ref. [4].

**Figure 3:**
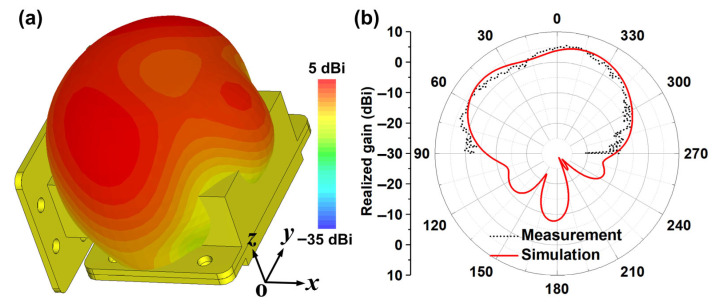
Comparison of the measured and simulated far-field results of the proposed sensor antenna. (**a**) Simulated 3D far-field pattern. (**b**) Simulated and measured 2D far-field pattern of *xoz* plane (H-plane).

**Figure 4:**
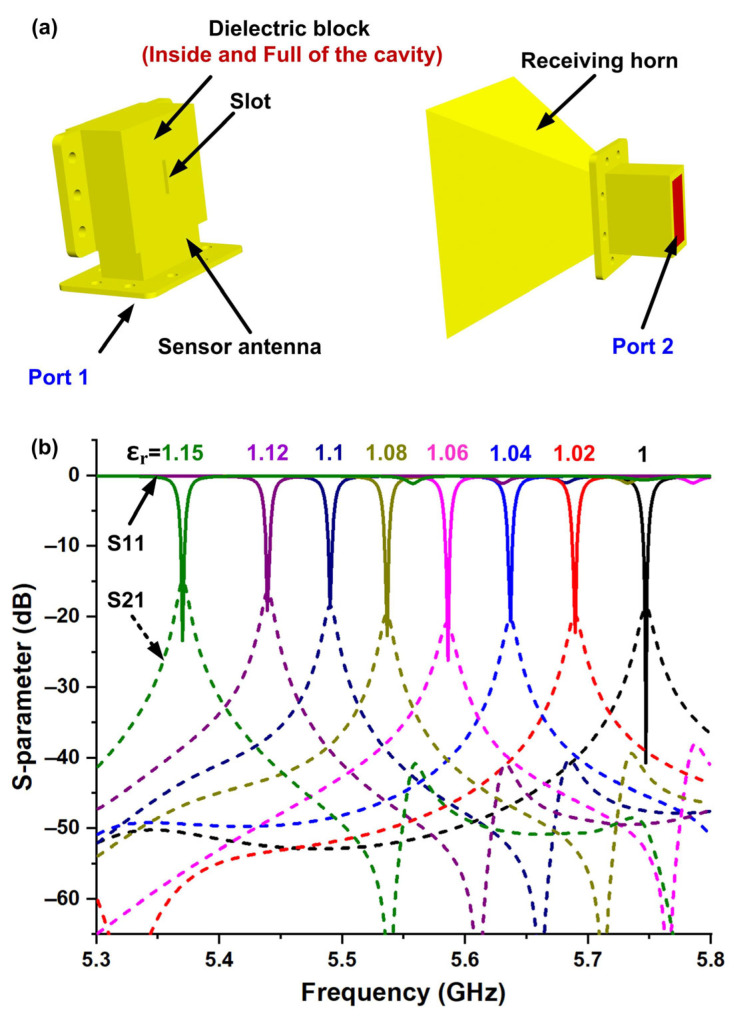
Simulation to verify the sensing capability of materials with different dielectric constants. (**a**) Simulation setup. (**b**) Simulated S-parameters.

Figure 3a,b show the simulated and measured far-field patterns of the designed sensing antenna at its resonant frequency. Figure 3a presents the simulated 3D far-field radiation pattern, indicating good broadside radiation and wide beam coverage. Figure 3b gives the simulated and measured 2D far-field radiation patterns in the *xoz* plane (i.e., H-plane), where the results are in good agreement, and the antenna’s peak gain is 5 dBi. In the CST software version 2024, the configuration for the non-contact sensing system is illustrated in Figure 4a. The sensing antenna and receiving horn antenna are positioned at opposite ends, with their separation distance set to 27 cm. This distance is strategically designed to balance computational efficiency and non-contact requirements. When electromagnetic signals are fed into the sensing antenna, two key parameters are acquired: the reflection coefficient (S11) at Port 1 and the transmission coefficient (S21) at the receiving horn antenna’s port (Port 2), which captures the wireless signal radiated through the antenna’s slot structure. To evaluate the sensing antenna’s capability to distinguish materials with varying dielectric properties, simulation models incorporate test dielectric blocks within the antenna’s cavity. These blocks match the cavity’s internal dimensions. As depicted in Figure 4b, when the relative dielectric constant of the inserted block increases from 1 to 1.15, the operating frequency of the antenna decreases from 5.75 GHz to 5.37 GHz accordingly, and the change process maintains a good linearity. In this way, the designed sensor antenna has a definite wireless sensing capability since the resonance peak can be obtained both from the reflection port (Port 1, S11) and from the receiving horn (Port 2, S21) in the non-contact form.

**Figure 5:**
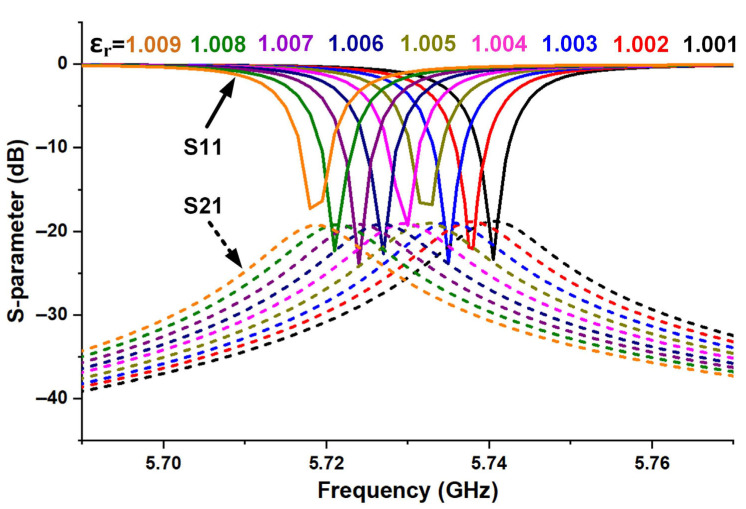
Simulated S-parameters of the sensing antenna with dielectric constants ranging from 1.001 to 1.009.

**Figure 6:**
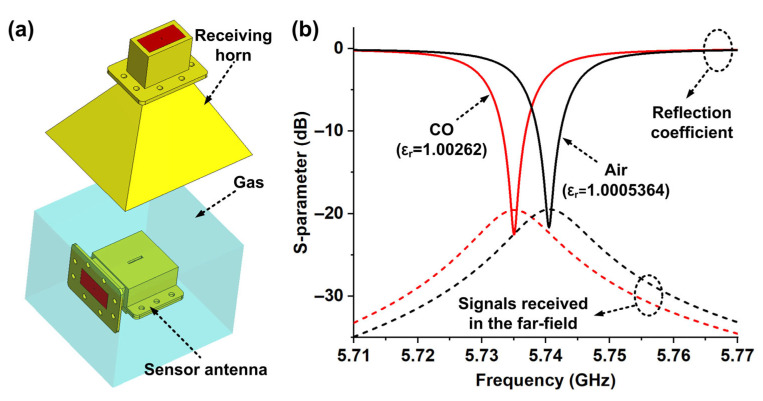
Simulation to verify the CO gas sensing capability. (**a**) Simulation setup. (**b**) Simulated S-parameters of air and CO, respectively.

The simulation results in Figure 4b demonstrate another remarkable feature of the designed sensor antenna, i.e., it is capable of detecting materials with ultra-low dielectric constants. Therefore, it is necessary to use gases that naturally have low dielectric constant properties as experimental samples. However, gas detection requires a dielectric constant resolution of less than 0.1%. Based on this, here we supplement the simulation results of dielectric constants ranging from 1.001 to 1.009. As shown in Figure 5, obvious changes in the resonant frequency of the sensing antenna are observed, indicating effective discrimination capability for gas dielectric constants. Figure 6 shows the effect of the designed sensor in distinguishing CO gas from air, with the corresponding simulation results provided. The relative dielectric constant of pure CO gas is 1.00262 at the standard atmospheric pressure (101,325 pa) and 20 °C, and that for air is 1.0005364 under the same conditions. As a result, the resonant frequency of the sensor changed significantly when the designed antenna was in each of the above environments full of the two gases, respectively. As shown in Figure 6a, the gas is set to completely wrap around the sensing antenna with the dimensions of 160 × 160 × 126.5 mm^3^ in the simulation. As indicated by the spectrums of Figure 6b, the resonance frequencies of the signals received by the receiving horn at a distance of 27 cm from the sensing antenna are 5.7405 GHz and 5.7351 GHz when the gases around the antenna are air and CO, respectively.

Sensing FoM is widely used to synthetically judge the sensitivity and detectability of sensors, and the calculation is written as (1) and (2), where the frequency shift Δ*f* is relative to the change in the dielectric constant *ε* (from *ε*_1_ to *ε*_2_, *ε*_1_ > *ε*_2_) [24,25,26].

(1) $$\mathrm{Sensitivity}=\frac{∆f}{\varepsilon_{1}-\varepsilon_{2}}$$

(2) $$\mathrm{Sensing}\;\mathrm{FoM}=\frac{\mathrm{Sensitivity}}{∆f_{3\mathrm{dB}}}$$

## 3. Results

We would like to emphasize that the designed sensing antenna is envisioned in the application scenario to be placed in a space where the gas to be measured is present, and the interior of the antenna is naturally filled with the gas to be measured due to the presence of the slot. Since the gas to be measured may be hazardous, only the transmitting end consisting of the sensing antenna and a simple swept EM wave generator is placed in the space to be measured at this point, while the receiving end can be in a safe position. This design is able to remotely monitor the dielectric constant of gases in the space to be measured by real-time sensing of spectral changes through the receiving antenna at a far-field distance based on the signals sent from the sensing antenna at the receiving end. Currently, transmission sensors and existing sensing antennas do not allow for this.

**Figure 7:**
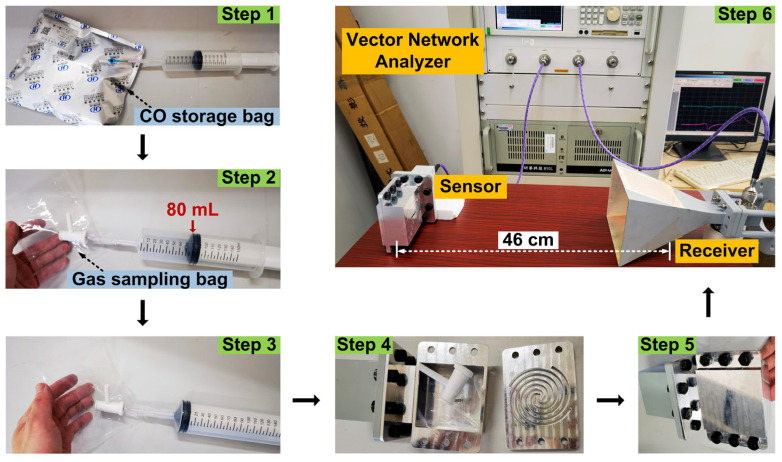
Experiment setup and test process.

However, validating the process exactly as described above would be costly and unaffordable to us. Therefore, in order to confirm the gas detection capability of the proposed sensing antenna in the laboratory, we constructed a remote CO gas sensor monitoring system as shown in Figure 7. The proposed sensing antenna and a horn antenna are 46 cm apart and connected to two ports of a vector network analyzer, respectively. The detailed experimental procedure is as follows:

Step 1: Use a syringe to draw 80 mL of CO gas from the CO gas storage bag.

Step 2: Take a 100 mL gas sampling bag and connect it to the syringe containing 80 mL of CO gas.

Step 3: Fill the gas sampling bag with all 80 mL of CO gas.

Step 4: Place the gas sampling bag containing 80 mL of CO gas into the cavity of the sensing antenna.

Step 5: Finish assembling the sensor cavity antenna.

Step 6: Connect the combined sensing antenna to the test system consisting of the vector network analyzer and the receiving antenna.

Step 7: Exhaust CO gas, fill with an equal amount of air, and retest.

Note 1: The above process was accomplished in an unsealed laboratory at a room temperature of 18 °C, i.e., the laboratory atmospheric pressure was approximated as standard atmospheric pressure.

Note 2: The purity of CO used in the experiment was 99%, which was produced by Dalian Kerui Gas Company (Dalian, China).

Note 3: The 100 mL single-valve gas sampling bag used in the experiment was produced by Ningbo Hongpu Experimental Technology Co. (Ningbo, China). The bag body and valve were made of Teflon materials.

It should be clarified that the above operation process of opening the cavity of the sensing antenna and placing the gas sampling bag, i.e., steps 1 to 5, is not required in the practical application scenario. This is because the gas in the space to be measured will automatically enter the interior of the cavity through the slot of the antenna, thus greatly simplifying the operation.

**Figure 8:**
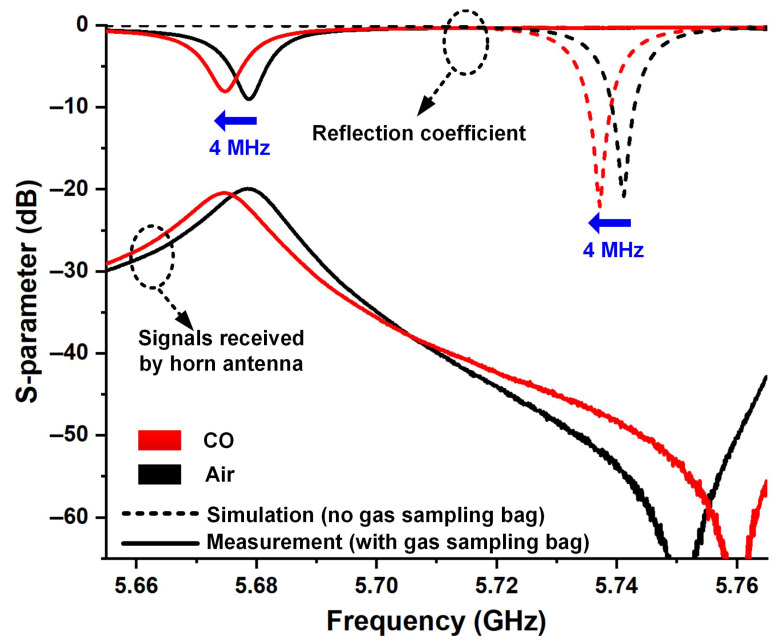
The measured spectrums of the proposed wireless sensor monitoring system.

Figure 8 gives the measured spectrums of the proposed remote sensor monitoring system, along with its approximate simulation results. It should be noted that in the simulation, the gas to be measured is designed as a regular rectangle with dimensions 80 × 45 × 22.2 mm^3^ and does not contain the gas sampling bag. As shown by dashed lines in Figure 8, the simulated resonance points are located at 5.7412 GHz and 5.7372 GHz when the cavity is filled with air and CO, respectively, and the simulated sensitivity and sensing FoM calculated from (1) and (2) are 3.842 GHz and 171.8 RIU^−1^. The measured S-parameters when there is air inside gas sampling bag in the sensing antenna are shown as black solid lines in Figure 8, where the line at the bottom is the signals received by the horn with the peak located at 5.6788 GHz. The red solid line in Figure 7 shows the measured S-parameters of the system when the gas sampling bag in the resonant cavity is filled with CO, with the resonance peak shifted downward to 5.6748 GHz. Therefore, the measured sensing FoM of this sensing antenna can be calculated as 3.842 GHz and 183.2 RIU^−1^, which is close to the simulation results. However, there are two main changes in the measured S-parameters compared to the simulation results; one is the overall shift of the resonance points toward lower frequencies, and the other is the change in the resonance depth, indicating an increase in antenna loss. We believe that both of them are related to the gas sampling bag that cannot be set in the simulation. This is because the sampling bag is made of Teflon, a material with a dielectric constant of 1.8–2.2 and a dielectric loss of 0.004. Therefore, in the experiment, these will increase the equivalent dielectric constant of the sample to be tested and introduce additional dielectric losses, which will result in the measured resonant frequencies being lower than those in the simulation and the measured resonant depth being shallower than that in the simulation, respectively.

Although the experiments confirm that the proposed method can discriminate CO from air, it should be noted that the sample used in this study was 100% pure CO gas. In fact, when CO and air are mixed in different proportions, the resonant frequency of the sensing antenna will change accordingly due to the different equivalent dielectric constants of the mixed gases. Therefore, the proposed sensing system theoretically also has corresponding discrimination capabilities. However, considering the complexity of experiments, these experimental conditions were limited, and further verification may be conducted in follow-up work.

## 4. Conclusions

In conclusion, we propose a sensing antenna for CO gas sensor monitoring applications. Operating in the microwave frequency band, this design features high sensitivity, high precision, and high reliability. The hybrid mode of cavity resonance and SLSP resonance enables the sensing antenna to achieve a high measurement Q-factor of 474.3 and a sensing FoM of 183.2 RIU^−1^. The experimental results demonstrate that this sensing antenna with the all-metal construction, high Q-factor, and high sensing FoM can be used for remote CO gas detection. Compared with other CO detection schemes, it may better adapt to harsh environments such as high temperature and high humidity.

## Data Availability

The data that support the findings of this study are available from the corresponding author upon reasonable request.
